# Supplementary material for: Morphological, Molecular, and Nutritional Characterisation of the Globe Artichoke Landrace “Carciofo Ortano”
Source: Plants (Basel). 2023 Apr 29;12(9):1844. doi: 10.3390/plants12091844 (PMC10180589; doi:10.3390/plants12091844)
Supplement: Supplementary file 1 [file plants-12-01844-s001.zip › plants-2274287-supplementary.pdf]

Supplementary materials

Table S1. *List of the 73 artichoke plants selected from the 20 smallholdings and family gardens (F1-20) located in the district of Orte Municipality and identified with the name of the keeper farmer. The number of plants (P) selected in each artichoke planting were indicated.*

1	Antonini Gervasio	F1 P1-2
2	Antonini Mario	F2 P1
3	Calderari Giorgio	F3 P1-3
4	Cecconi Luciana	F4 P1-13
5	Del Gelsomino Sergio	F5 P1-8
6	Scuola Orte	F6 P1-2
7	Manzara Mattia	F7 P1-5
8	Marzoli Franco	F8 P1-5
9	Nicoletti Ottavio	F9 P1
10	Paolessi Claudio	F10 P1-3
11	Paolessi Riccardo	F11 P1
12	Riccardi Sabrina	F12 P1-2
13	Tofone Franco	F13 P1-2
14	Sensini Franco	F14 P1-2
15	Piersanti Giovanni	F15 P1-3
16	Pompei Claudio	F16 P1-2
17	Amintore Massarelli	F17 P1-6
18	Tilesi Sandro	F18 P1-4
19	Ridolfi Marco	F19 P1-4
20	Tofone Erino	F20 P1-4

Table S2. *List of the 17 accessions of landraces/clones belonging to the four varietal types cultivated in Italy ("Romanesco", "Violetto", "Spinoso" and "Catanese") used as reference genotypes.*

Accession name	Varietal typology	Origin/area of cultivation	Quality labels
1) Campagnano	Romanesco	Landrace cultivated in the coastal area of Lazio[9]	PGI "Carciofo Romanesco del Lazio"
2) Castellammare	Romanesco	Landrace cultivated in the coastal area of Lazio[9]	PGI "Carciofo Romanesco del Lazio"
3) Castellammare Sezze	Romanesco	Landrace cultivated in the coastal area of Lazio[9]	PGI "Carciofo Romanesco del Lazio"
4) C3	Romanesco	Clone selected from the Castellamare landrace[29]	PGI "Carciofo Romanesco del Lazio"
5) Raffaello	Romanesco	Clone selected from the Castellamare landrace[29]	PGI "Carciofo Romanesco del Lazio"
6) Michelangelo	Romanesco	Clone selected from the C3[29]	PGI "Carciofo Romanesco del Lazio"
7) Donatello	Romanesco	Clone selected from the Campagnano landrace[29]	
8) Grato 1	Romanesco	Clone selected from plants obtained by cross-pollination of clones belonging to the Castellammare, Campagnano and Violetto di Toscana landraces[29]	
9) Leonardo	Romanesco	Clone selected from Grato 1[29]	PGI "Carciofo Romanesco del Lazio"
10) Montelupone	Romanesco	Landrace cultivated in the province of Macerata (Marche)[9]	Slow Food Presidium
11) Terom	Violetto	Clone selected from the Violetto di Toscana landrace[29]	
12) Tardivo di Pesaro	Violetto	Landrace cultivated in the province of Pesaro/Urbino (Marche)[30]	
13) Catanese	Catanese	Landrace cultivated in the province of Catania (Sicily)[31]	
14) Brindisino	Catanese	Landrace cultivated in the province of Brindisi (Apulia)[32]	PGI "Carciofo Brindisino"
15) Spinoso Sardo	Spinoso	Landrace cultivated in the Sardinia region[33]	PDO "Carciofo Spinoso di Sardegna"
16) Bianco Ostuni	Off type	Landrace cultivated in the province of Brindisi (Apulia)[34]	
17) Carciofino di Pontecorvo	Off type	Landrace cultivated in the province of Latina (Lazio)	

Table S3. *Characteristics of the 12 SSR markers used in this study.*

SSR code	Repeat motif	Forward e Reverse primers	LG	Genebank No.	Ta (°C)	Dye color	References
CDAT_02	$(TGA)_5T$	F: GCGCTGCAAAGTTATTT<br>R: TGAGTTCTTAATCATCATCTTC	LG1	A86530	50	FAM	Acquadro et al. [35]
CELMS_08	$(TC)_{22}(TC)_8(TC)_{10}$	F: TTTACAAACTTCCCCTTTCCAC<br>R: ACAATACAGATACACGCTCTCCA	LG1	EU744924	57	JOE	Acquadro et al. [38]
CDAT_04	$(TC)_{11}$	F: CCGAACAGTATGTCCTGA<br>R: AGTCATGTACCTGCTCCA	-	X69193.1	54	TAMRA	Acquadro et al. [35]
Cmal_06	$(CT)_{13}$	F: AAGTAATCATTTTCACAAGGTC<br>R: CCGATATAGATGAACCTAGC	LG1	AY601785<br>AY601786	54	FAM	Acquadro et al. [36]
CLIB_02	$(CA)_{11}$	F: AGTCATGTACCTGCTCCA<br>R: CATTTGAGCAGGCTTGGTTC	LG12	AF527029	57	JOE	Acquadro et al. [35]
CELMS_05	$(CT)_{22}(CAG)_4$	F: CCCACCTTCTTATCCCATCA<br>R: TGGACGTCTGTTTCCTCCTC	LG1	EU744921<br>EU744922	58	TAMRA	Acquadro et al. [38]
Cmal_08	$(CT)_{18}$	F: AGTGTTGAGCAACGATATGG<br>R: AATCCAATTCAACAAAATCTAC	LG1	AY601791<br>AY601792	53	FAM	Acquadro et al. [36]
CDAT_01	$(TGA)_5T.A(TGA)_5T.(GTG)_5$	F: CATTTGAGCAGGCTTGGTTC<br>R: CGGAATCATGCTAACCATA	LG1	A86530	52	JOE	Acquadro et al. [35]
CELMS_40	$(ATC)_4(ATC)_{16}(TCT)_9$	F: TGGATTAAGGCACACACTGAAC<br>R: TGATGATAACAAAGGAGGGGAT	LG1	EU744956	57	TAMRA	Acquadro et al. [38]
Cmal_07	$(AG)_7$	F: AACCTGGTCGATGAATAATG<br>R: GGTCTTGGTCTTCATGTCTC	-	AY601787<br>AY601788	55	FAM	Acquadro et al. [36]
CMAFLP_18	$(CA)_8(TA)_6(CA)_5(TA)_4(GT)_5$	F: AAGTGTTGCATAATAACTTACC<br>R: CCGAACAAATTGCTTACAA	-	AY795836<br>AY795837	52	JOE	Acquadro et al. [37]
CsPal_03	$(TA)_5CA(TA)_2(CATA)_4(CA)_6$	F: ACTTGCCTTCTCTTGTGCCTACCT<br>R: TCCGCAACCATTCTCTTCACCTCA	LG1	AM497826	62	TAMRA	Sonnante et al. [39]

Table S4. *Characteristics of the 10 ISSR markers used in this study.*

ISSR code	Sequence 5'-3'	Ta (°C)	References
ISSR_24	$(GT)_8YC$	55	Ciaffi et al. [40]
ISSR_28	$(GT)_8RA$	55	Ciaffi et al. [40]
UBC_840	$(GA)_8YT$	55	UBC primer dataset
ISSR_823	$(TC)_8C$	55	UBC primer dataset
UBC_836	$(AG)_8YA$	55	UBC primer dataset
UBC_837	$(TA)_8RT$	55	UBC primer dataset
UBC_848	$(CA)_8RG$	55	UBC primer dataset
UBC_853	$(TC)_8RT$	55	UBC primer dataset
UBC_855	$(AC)_8YT$	55	UBC primer dataset
UBC_857	$(AC)_8YG$	55	UBC primer dataset

Y=T/C;
R=G/A

Table S5. *Forty-three morphological descriptors used in this study for the characterization of representative genotypes of the "Carciofo Ortano" landrace and the four landraces/clones used as reference genotypes.*

Plant	PH	Plant height (cm)
	MSL	Main stem lenght (cm)
	DHL	Distance between the youngest leaf on the main floral stem and the central flower-head (cm)
	MSD	Main stem diameter (cm)
	TNH	Total number of heads
	Y	Total production (g)
Leaf	Leafatt	Leaf attitude
	Spines	Spines
	Linc	Leaf incision
	ShTip	Shape of leaf tip
	IntGreen	Intensity of leaf green colour
	HueGreen	Hue of leaf green colour
	GreyHue	Intensity of leaf grey hue
	Hair	Leaf hairness
	Blistering	Leaf blistering
	Anthocpet	Anthocyanin coloration at base of petiole
Heads	DCH	Diameter of the central heads (cm)
	LCH	Lenght central heads (cm)
	WCH	Weight of the central heads (g)
	L/D ratio	Length/diameter ratio for the central head shape
	LH1	Lenght of the first lateral head on the lateral shoots (cm)
	DH1	Diameter of the first order heads on the lateral shoots (cm)
	WH1	Weight of the primary heads (g)
	L/D ratio 1	Length/diameter ratio for the first lateral head shape
	THA	Time of central heads appareance (days)
	THM	Time of head maturity (days)
	HeadShape	Central flower head: shape in longitudinal section
	ApexShape	Central flower head: shape of apex
	ShapeHead1	Shape of first flower head on lateral shoot
	OpenHead1	First flower head level of openness
Outer bract	ShapeBract	Main shape of outer bract
	ApexBract	Shape of bract apex
	DepthBract	Depth of incision of outer bract
	ColBract	Color of outer bract
	HueColBract	Hue of secondary color of outer bract
	CurvBract	Bract tip curvature
	SizeSpine	Size of spine of outer bract
	Mucron	Mucron
	AnthoHeadBract	Anthocyanin coloration of central flower head inner bracts
	DensityBracts	Density of inner bracts
Receptacle	ShapeRec	Shape of receptacle in longitudinal section
	RecD	Diameter of receptacle (cm)
	RecT	Receptacle thickness (cm)

Table S6. *SSR rare and private alleles detected in the 90artichoke genotypes in homozygous (Hom) or heterozygous (Het) status. The asterisk indicates the private allele.*

Locus	Allele	Frequency	Genotype	Farmer	Status
CDAT_02	168*	0.01	Bianco Ostuni		Hom
CELMS_08	247*	0.01	Bianco Ostuni		Het
CDAT_04	403	0.044	F9 P1	Nicoletti Ottavio	Het
			Tardivo Pesaro		Het
			Brindisino		Het
			Catanese		Het
	425	0.044	Terom		Het
			Grato 1		Het
			Leonardo		Het
			F1 P1	Antonini Gervasio	Hom
Cmal_06	118	0.022	F9 P1	Nicoletti Ottavio	Het
			Tardivo Pesaro		Het
CELMS_05	300	0.022	Brindisino		Het
			Catanese		Het
	305*	0.01	Bianco Ostuni		Het
	316	0.022	F9 P1	Nicoletti Ottavio	Hom
			Tardivo Pesaro		Hom
	318	0.044	F1 P1	Antonini Gervasio	Hom
			Terom		Het
			Grato 1		Het
			Leonardo		Het
	340	0.033	Bianco Ostuni		Het
			Pontecorvo		Hom
			Spinoso Sardo		Hom
Cmal_08	122	0.033	F1 P1	Antonini Gervasio	Het
			Grato 1		Het
			Leonardo		Het
	144	0.022	F18 P2	Tilesi Sandro	Het
			F18 P3	Tilesi Sandro	Het
CDAT_01	197	0.044	Brindisino		Het
			Catanese		Het
			Pontecorvo		Het
			Spinoso Sardo		Het
	199*	0.01	Bianco Ostuni		Het
	201	0.044	F1 P1	Antonini Gervasio	Het
			Terom		Het
			Grato 1		Het
			Leonardo		Het
	217*	0.01	Bianco Ostuni		Het
CELMS_40	377	0.044	Brindisino		Hom
			Catanese		Hom
			Pontecorvo		Hom
			Spinoso Sardo		Hom
	389	0.044	F8 P1	Marzoli Franco	Het
			F8 P3	Marzoli Franco	Het
			F8 P4	Marzoli Franco	Het
			F8 P5	Marzoli Franco	Het
	418	0.033	F1 P2	Antonini Gervasio	Hom
			F6 P1	Scuola Orte	Hom
			F6 P2	Scuola Orte	Hom
CsPal_03	339	0.033	F1 P2	Antonini Gervasio	Hom
			F6 P1	Scuola Orte	Hom
			F6 P2	Scuola Orte	Hom

Table S7. *ISSR rare and private bands (alleles) detected in the 90 artichoke genotypes. The asterisk indicates the private allele.*

Locus	Frequency	Genotype	Farmer
ISSR_24/2	0.033	F1 P2	Antonini Gervasio
		F6 P1	Scuola Orte
		F6 P2	Scuola Orte
ISSR_24/3	0.022	Brindisino	
		Catanese	
ISSR_24/5	0.022	F12 P1	Riccardi Sabrina
		F13 P2	Tofone Franco
ISSR_24/13	0.022	F12 P1	Riccardi Sabrina
		F13 P2	Tofone Franco
ISSR_28/18	0.044	F18 P1	Tilesi Sandro
		F18 P2	Tilesi Sandro
		F18 P3	Tilesi Sandro
		F18 P4	Tilesi Sandro
ISSR_28/20	0.044	F19 P1	Ridolfi Marco
		F19 P2	Ridolfi Marco
		F19 P3	Ridolfi Marco
		F19 P4	Ridolfi Marco
ISSR_28/21	0.033	F18 P2	Tilesi Sandro
		F18 P3	Tilesi Sandro
		F18 P4	Tilesi Sandro
ISSR_28/22	0.044	F18 P1	Tilesi Sandro
		F18 P2	Tilesi Sandro
		F18 P3	Tilesi Sandro
		F18 P4	Tilesi Sandro
ISSR_28/23	0.033	F1 P2	Antonini Gervasio
		F6 P1	Scuola Orte
		F6 P2	Scuola Orte
ISSR_28/25	0.022	F1 P1	Antonini Gervasio
		Terom	
ISSR_28/26	0.22	F18 P3	Tilesi Sandro
		F18 P4	Tilesi Sandro
ISSR_28/27*	0.01	F18 P4	Tilesi Sandro
UBC_840/29	0.033	F1 P2	Antonini Gervasio
		F6 P1	Scuola Orte
		F6 P2	Scuola Orte
UBC_840/32	0.044	F19 P1	Ridolfi Marco
		F19 P2	Ridolfi Marco
		F19 P3	Ridolfi Marco
		F19 P4	Ridolfi Marco
UBC_840/33	0.044	F19 P1	Ridolfi Marco
		F19 P2	Ridolfi Marco
		F19 P3	Ridolfi Marco
		F19 P4	Ridolfi Marco
UBC_840/34*	0.01	Bianco Ostuni	
UBC_840/36	0.022	F19 P1	Ridolfi Marco
		F19 P2	Ridolfi Marco
UBC_840/43*	0.01	Montelupone	
UBC_848/52*	0.01	F1 P2	Antonini Gervasio

Table S7. *(Continued)*

UBC_855/60	0.022	Brindisino	
		Catanese	
UBC_855/61	0.044	F1 P1	Antonini Gervasio
		Terom	
		Grato_1	
		Leonardo	
UBC_855/65*	0.01	Bianco Ostuni	
UBC_857/72	0.044	F17 P4	Massarelli Amintore
		Castellammare	
		Sezze	
		Raffaello	
UBC_857/73	0.044	F1 P1	Antonini Gervasio
		Terom	
		Grato_1	
		Leonardo	

Table S8. *Posterior membership coefficient (Q) following STRUCTURE analysis with K= 2.*

**Genotype**	**Group 1**	**Group 2**
Sezze	0.998	0.002
Raffaello	0.999	0.001
Castellammare	0.998	0.002
Michelangelo	0.998	0.002
C3	0.999	0.001
F20 P3	0.999	0.001
F20 P2	0.999	0.001
Donatello	0.998	0.002
Campagnano	0.998	0.002
F13 P2	0.998	0.002
F12 P1	0.998	0.002
F20 P4	0.998	0.002
F20 P1	0.998	0.002
F17 P4	0.997	0.003
F17 P3	0.998	0.002
F17 P1	0.989	0.011
F4 P1	0.997	0.003
F4 P10	0.997	0.003
F4 P11	0.997	0.003
F4 P12	0.997	0.003
F4 P8	0.997	0.003
F4 P9	0.997	0.003
F4 P2	0.997	0.003
F4 P4	0.997	0.003
F4 P5	0.997	0.003
F4 P6	0.997	0.003
F4 P13	0.989	0.011
F4 P3	0.997	0.003
F19 P4	0.998	0.002
F19 P2	0.995	0.005
F19 P1	0.995	0.005
F19 P3	0.995	0.005
F17 P5	0.973	0.027
F17 P6	0.893	0.107
F17 P2	0.796	0.204
F10 P1	0.998	0.002
F3 P1	0.998	0.002
F3 P2	0.998	0.002
F3 P3	0.998	0.002
F10 P2	0.998	0.002
F10 P3	0.998	0.002
F8 P2	0.857	0.143
F12 P2	0.984	0.016
F4 P7	0.990	0.010
Montelupone	0.228	0.772
F13 P1	0.223	0.777
F2 P1	0.191	0.809
F8 P5	0.081	0.919
F8 P4	0.059	0.941
F8 P3	0.059	0.941
F8 P1	0.058	0.942

Table S8. *(Continued)*

F11 P1	0.021	0.979
F7 P2	0.006	0.994
F7 P4	0.006	0.994
F7 P3	0.005	0.995
F14 P1	0.002	0.998
F14 P2	0.002	0.998
F15 P1	0.002	0.998
F15 P3	0.002	0.998
F7 P1	0.002	0.998
F7 P5	0.002	0.998
F16 P1	0.002	0.998
F16 P2	0.002	0.998
F15 P2	0.002	0.998
F5 P1	0.002	0.998
F5 P2	0.002	0.998
F5 P3	0.002	0.998
F5 P4	0.002	0.998
F5 P5	0.002	0.998
F5 P6	0.002	0.998
F5 P7	0.002	0.998
F5 P8	0.002	0.998

Table S9. *SSR private alleles detected in Orte 1 and Orte 2 populations.*

Locus	Allele	Population	Frequency	Genotype	Genetic group
CELMS_08	241	Orte 2	0.30	F5 P1-8	GGR 14
CDAT_04	405	Orte 2	0.04	F13 P1	Sep2
CDAT_04	411	Orte 2	0.04	F13 P1	Sep2
Cmal_06	112	Orte 2	0.04	F13 P1	Sep2
CELMS_40	389	Orte 2	0.15	F8 P1-3-4-5	GGR 10
CELMS_40	422	Orte 2	0.15	F8 P1-3-4-5	GGR 10
CELMS_40	424	Orte 2	0.85		
CDAT_02	182	Orte 2	1.00		
CELMS_05	338	Orte 1	1.00		
CELMS_05	329	Orte 2	1.00		
CDAT_01	213	Orte 1	1.00		
CDAT_01	207	Orte 2	1.00		
CELMS_40	379	Orte 1	1.00		
CsPal_03	335	Orte 1	1.00		
CsPal_03	331	Orte 2	1.00		

Table S10. *ISSR private alleles detected in Orte 1 and Orte 2 populations.*

Locus	Population	Frequency	Accessions name	Genetic group
ISSR24/5	Orte 1	0.05	F12 P1; F13 P2	GGR2
ISSR24/10	Orte 2	0.04	F11 P1	Sep4
ISSR24/12	Orte 1	0.16	F12 P1; F13 P2; F20 P1-4	GGR2/3
ISSR24/13	Orte 1	0.05	F12 P1; F13 P2	GGR2
ISSR24/14	Orte 2	0.30	F5 P1-8	GGR14
ISSR24/16	Orte 1	0.16	F3 P1-3; F10 P1-3	GGR8
ISSR28/20	Orte 1	0.11	F19 P1-4	GGR6
UBC840/32	Orte 1	0.11	F19 P1-4	GGR6
UBC840/33	Orte 1	0.11	F19 P1-4	GGR6
UBC840/36	Orte 1	0.05	F19 P1-2	GGR6
UBC840/37	Orte 1	0.16	F12 P1; F13 P2; F20 P1-4	GGR2/3
UBC840/42	Orte 2	1.00	Orte 2	
UBC848/46	Orte 1	0.51	F4 P1-13; F19 P1-4; F20 P1-3	GGR1/3/5/6
UBC848/51	Orte 2	0.15	F8 P1/3-5	GGR10
UBC855/55	Orte 1	0.30	F4 P1-2/P4-12	GGR5
UBC855/57	Orte 1	0.76	F3 P1-3; F4 P1-6/8-13; F10 P1-3; F12 P1; F13 P2; F17 P1-3; F19 P4; F20 P1-4	GGR1-8
UBC855/63	Orte 2	0.67	F5 P1-8; F7 P1/5; F11 P1; F14 P1-2; F15 P1-3; F16 P1-2	GGR12-14
UBC857/67	Orte 1	0.08	F17 P1/3-4	GGR4
UBC857/69	Orte 1	0.78	F3 P1-3; F4 P1-6/8-13; Sep1; F10 P1-3; F12 P1; F13 P2; F17 P1-3; F19 P4; F20 P1-4	GGR1-8
UBC857/72	Orte 1	0.03	F17 P6	GGR7
UBC857/75	Orte 2	0.67	F5 P1-8; F7 P1/5; F11 P1; F14 P1-2; F15 P1-3; F16 P1-2	GGR12-14

Table S11. *Morphological qualitative traits retrieved by using UPOV descriptors for representative genotypes of Orte 1 (GGR4, GGR5 and GGR8) and Orte 2 (GGR10, GGR11 and GGR12) populations and for the four landraces/clones belonging to the "Romanesco" type used as reference genotypes ("Campagnano", "Castellammare", "C3", and "Grato 1"). In red are shown the 10 characters for which differences were found among the examined genotypes.*

QUALITATIVE MORPHOLOGICAL TRAITS	Campagnano	Castellammare	C3	Grato 1	Orte1-GGR4 F17	Orte1-GGR5 F4	Orte1-GGR8 F3	Orte2-GGR12 F7	Orte2-GGR11 F7	Orte2-GGR10 F8
Leaf attitude	Semi-erect	Semi-erect	Semi-erect	Semi-erect	Semi-erect	Semi-erect	Semi-erect	Semi-erect	Semi-erect	Semi-erect
Spines	Absent	Absent	Absent	Absent	Absent	Absent	Absent	Absent	Absent	Absent
Leaf incision	Present	Present	Present	Present	Present	Present	Present	Present	Present	Present
Shape of leaf tip	Acute	Acute	Acute	Acute	Acute	Acute	Acute	Acute	Acute	Acute
Intensity of leaf green colour	Medium	Medium	Medium	Medium	Medium	Medium	Medium	Medium	Medium	Medium
Hue of leaf green colour	Gray green	Gray green	Gray green	Gray green	Gray green	Gray green	Gray green	Gray green	Gray green	Gray green
Intensity of leaf grey hue	Medium	Medium	Medium	Medium	Medium	Medium	Medium	Medium	Medium	Medium
Leaf hairness	Absent	Absent	Absent	Absent	Absent	Absent	Absent	Absent	Absent	Absent
Leaf blistering	Slight	Slight	Slight	Slight	Slight	Slight	Slight	Slight	Slight	Slight
Anthocyanin coloration at base of petiole	Absent	Absent	Absent	Absent	Absent	Absent	Absent	Absent	Absent	Absent
Central flower head: shape in longitudinal section	Rounded	Rounded	Rounded	Triangular	Rounded	Rounded	Rounded	Elliptical	Elliptical	Elliptical
Central flower head: shape of apex	Rounded	Rounded	Rounded	Rounded	Rounded	Rounded	Rounded	Flattened	Flattened	Flattened
Shape of first flower head on lateral shoot	Rounded	Rounded	Rounded	Triangular	Rounded	Rounded	Rounded	Elliptical	Elliptical	Elliptical
First flower head level of openness	Semi-open	Semi-open	Semi-open	Semi-open	Semi-open	Semi-open	Semi-open	Semi-open	Semi-open	Semi-open
Main shape of outer bract	As long as broad	As long as broad	As long as broad	Longer than broad	As long as broad	As long as broad	As long as broad	Broader than long	Broader than long	Broader than long
Shape of bract apex	Depressed	Depressed	Depressed	Depressed	Depressed	Depressed	Depressed	Depressed	Depressed	Depressed
Depth of incision of outer bract	Medium	Medium	Medium	Deep	Medium	Medium	Medium	Medium	Medium	Medium
Color of outer bract	Violet with green streaks	Violet with green streaks	Violet with green streaks	Mainly violet	Violet with green streaks	Violet with green streaks	Violet with green streaks	Violet with green streaks	Violet with green streaks	Violet with green streaks
Hue of secondary color of outer bract	Absent	Absent	Absent	Absent	Absent	Absent	Absent	Absent	Absent	Absent
Bract tip curvature	Absent	Absent	Absent	Absent	Absent	Absent	Absent	Absent	Absent	Absent
Size of spine of outer bract	Absent	Absent	Absent	Absent	Absent	Absent	Absent	Absent	Absent	Absent
Mucron	Absent	Present	Absent	Present	Absent	Absent	Absent	Absent	Absent	Absent
Anthocyanin coloration of central flower head inner bracts	Slight	Slight	Slight	Strong	Slight	Slight	Slight	Slight	Slight	Slight
Density of inner bracts	Dense	Dense	Dense	Spaced	Dense	Dense	Dense	Dense	Dense	Dense
Shape of receptacle in longitudinal section	Slightly depressed	Slightly depressed	Slightly depressed	Slightly depressed	Slightly depressed	Slightly depressed	Slightly depressed	Depressed	Depressed	Depressed

Table S12. *Total eigenvalues, relative and cumulative proportion of total variance explained by each component and link of the first three PCs with the main morphological traits.*

**Component**	**Total eigenvalues**	**Variance explained (%)**	**Cumulative variance (%)**	**Larger correlation with main traits**
1	9,31	51,7	51,7	WCH, LCH, WH1, LH1, Y
2	4,26	23,7	75,4	LD and LD1 ratios, RecD, MSD
3	2,50	13,9	89,2	THA, THM, PH, MSL, DHL
4	0,91	5,1	94,3	
5	0,49	2,7	97,0	
6	0,34	1,9	99,0	
7	0,15	0,8	99,8	
8	0,03	0,1	99,9	
9	0,01	0,1	100,0	

Table S13. *Differences in eighteen morphological quantitative traits (means of six measurements) of the genotypes of Orte 1 (GGR4, GGR5 and GGR8) and Orte 2 (GGR10, GGR11 and GGR12) populations and the four landraces/clones belonging to the "Romanesco" type. ("Campagnano", "Castellammare", "C3" and "Grato 1"). Different letters indicate statistically significant differences among accessions at p ≤ 0.05 (ANOVA analysis, Tukey test); *** indicate significant differences at p ≤ 0.001; ** indicate significant differences at p ≤ 0.01; * indicates significant differences at p ≤ 0.05; ns indicate no significant differences. PH: Plant height (cm); MSL: Main stem length (cm); MSD: Main stem diameter (cm); DHL: Distance between the youngest leaf on the main floral stem and the central; flower-head (cm); LCH: Length central head (cm); DCH: Diameter of the central head (cm); L/D ratio: Length/Diameter ratio of central head (cm/cm); RECD: Diameter of receptacle (cm); WCH: Weight of the central head (g); LH1: Length of the first lateral head on the lateral shoots (cm); DH1: Diameter of the first order head on the lateral shoots (cm); L/D ratio1: Length/Diameter ratio of first lateral head (cm/cm); WH1: Weight of the primary head (g); TNH: Total number of heads; Y: Total production (g); THA: Time of central heads appearance (days); THM: Time of heads maturity (days). CV: coefficient of variation expressed as percentage.*

	PH	MSL	MSD	DHL	LCH	DCH	L/D ratio	RECD	RECT
**Campagnano**	66.833^{b}	57.833^{b}	2.316^{bc}	24.750^{ab}	90.000^{a}	9.500^{abc}	0.947^{b}	4.566^{ab}	1.090^{abc}
**Castellammare**	52.833^{d}	43.983^{e}	2.621^{a}	20.450^{c}	88.500^{a}	9.433^{abc}	0.938^{b}	4.666^{ab}	1.066^{abc}
**C3**	58.666^{cd}	48.983^{cd}	2.666^{a}	20.666^{c}	96.833^{a}	9.950^{ab}	0.973^{b}	4.983^{a}	1.150^{abc}
**Grato 1**	61.666^{bc}	52.066^{c}	2.200^{c}	22.916^{bc}	96.000^{a}	8.600^{c}	1.115^{a}	4.000^{b}	0.916^{bc}
**Orte1-GGR4 F17**	73.000^{a}	63.183^{a}	2.533^{ab}	27.000^{a}	98.166^{a}	10.216^{a}	0.960^{b}	5.150^{a}	1.266^{a}
**Orte1-GGR5 F4**	67.000^{b}	57.433^{b}	2.483^{ab}	23.166^{bc}	95.666^{a}	10.050^{ab}	0.953^{b}	4.983^{a}	1.083^{abc}
**Orte1-GGR8 F3**	74.333^{a}	64.833^{a}	2.316^{bc}	24.333^{ab}	95.000^{a}	10.000^{ab}	0.955^{b}	5.16^{a}	1.200^{ab}
**Orte2-GGR12 F7**	53.333^{d}	46.066^{de}	2.383^{bc}	20.000^{c}	72.666^{b}	9.033^{bc}	0.805^{c}	4.800^{a}	0.883^{c}
**Orte2-GGR11 F7**	54.500^{d}	45.350^{de}	2.403^{bc}	20.333^{c}	74.833^{b}	9.466^{abc}	0.790^{c}	4.850^{a}	1.000^{abc}
**Orte2-GGR10 F8**	54.000^{d}	46.600^{de}	2.400^{bc}	21.000^{c}	74.000^{b}	9.233^{abc}	0.801^{c}	4.716^{a}	0.933^{bc}
**CV**	13,368	14,597	5,947	10,497	11,724	5,351	10,823	7,099	11,922
p	***	***	***	***	***	***	***	***	**

	WCH	LH1	DH1	L/D ratio1	WH1	TNH	Y	THA	THM
**Campagnano**	260.558^{abc}	5.333^{a}	5.283^{ab}	1.011^{b}	81.240^{bc}	6.333^{a}	692.798^{bc}	102.333^{b}	123.166^{b}
**Castellammare**	245.468^{abc}	5.066^{ab}	5.083^{bc}	0.996^{b}	71.316^{c}	6.500^{a}	617.913^{cd}	95.000^{c}	112.333^{c}
**C3**	287.665^{ab}	5.350^{a}	5.433^{ab}	0.983^{b}	94.765^{a}	6.500^{a}	798.718^{a}	84.166^{d}	105.333^{d}
**Grato 1**	230.485^{bc}	5.350^{a}	4.566^{c}	1.173^{a}	76.138^{c}	5.833^{a}	597.956^{d}	103.833^{b}	123.500^{b}
**Orte1-GGR4 F17**	303.815^{a}	5.783^{a}	5.833^{a}	0.991^{b}	91.836^{ab}	6.166^{a}	768.151^{a}	105.000^{b}	124.333^{b}
**Orte1-GGR5 F4**	304.283^{a}	5.716^{a}	5.566^{ab}	1.028^{b}	93.245^{a}	6.166^{a}	785.028^{a}	105.666^{b}	124.000^{b}
**Orte1-GGR8 F3**	285.355^{ab}	5.366^{a}	5.283^{ab}	1.016^{b}	90.566^{ab}	5.833^{a}	724.255^{ab}	103.333^{b}	123.333^{b}
**Orte2-GGR12 F7**	231.573^{bc}	4.300^{c}	4.916^{bc}	0.876^{c}	72.033^{c}	6.333^{a}	589.273^{d}	118.000^{a}	138.000^{a}
**Orte2-GGR11 F7**	238.103^{bc}	4.533^{bc}	5.250^{ab}	0.865^{c}	74.323^{c}	6.333^{a}	608.093^{d}	118.000^{a}	137.000^{a}
**Orte2-GGR10 F8**	222.413^{c}	4.416^{bc}	5.000^{bc}	0.885^{c}	73.206^{c}	6.333^{a}	588.620^{d}	118.333^{a}	137.000^{a}
**CV**	12,159	10,337	6,807	9,305	11,829	3,825	12,769	10,301	8,504
p	***	***	***	***	***	ns	***	***	***

Table S14. *Mineral content expressed as mg 100 g^{-1} FW (means of six analyses) of the heads of the eight studied artichoke genotypes. Different letters indicate statistically significant differences among genotypes at p ≤ 0.05 (ANOVA analysis, Tukey test); *** indicate significant differences at p ≤ 0.001; ** indicate significant differences at p ≤ 0.01; * indicates significant differences at p ≤ 0.05; ns indicate no significant differences. CV: coefficient of variation expressed as percentage.*

	**K**	**Na**	**Ca**	**P**	**Mg**	**Fe**	**Zn**
**Campagnano**	320.50^{a}	104.00^{a}	87.83^{a}	70.5^{a}	46.67^{a}	1.10^{c}	0.62^{a}
**Castellammare**	318.33^{ab}	100.33^{ab}	67.00^{c}	66.83^{ab}	45.33^{a}	1.13^{bc}	0.63^{a}
**C3**	315.00^{ab}	98.33^{b}	63.17^{cd}	68.50^{ab}	43.33^{ab}	1.08^{c}	0.65^{a}
**Grato 1**	306.33^{b}	89.00^{d}	72.33^{b}	67.33^{ab}	43.67^{ab}	1.15^{bc}	0.72^{a}
**Orte1-GGR4 F17**	312.33^{ab}	96.67^{bc}	91.00^{a}	68.50^{ab}	42.00^{ab}	1.10^{c}	0.70^{a}
**Orte1-GGR5 F4**	307.00^{b}	95.33^{bc}	90.00^{a}	69.17^{ab}	42.50^{ab}	1.08^{c}	0.67^{a}
**Orte2-GGR10 F8**	292.17^{c}	91.17^{cd}	58.67^{d}	65.67^{ab}	39.33^{b}	1.28^{ab}	0.72^{a}
**Orte2-GGR11 F7**	290.00^{c}	89.67^{d}	58.00^{d}	64.33^{b}	39.67^{b}	1.33^{a}	0.73^{a}
**CV**	3.70	5.59	19.20	2.94	5.92	8.28	6.39
p	***	***	***	*	**	***	ns

Table S15. *Total flavonoids content (TFC) expressed as mg RUE g^{-1} DW and total polyphenols content (TPC) expressed as mg GAE g^{-1} DW of the heads of the eight studied artichoke genotypes. The reported values are the means of six analyses. Different letters indicate statistically significant differences among genotypes at $p \leq 0.05$ (ANOVA analysis, Tukey test); *** indicate significant differences at $p \leq 0.001$. CV = coefficient of variation expressed as percentage.*

	**TFC** mg RUE g^{-1} DW	**TPC** mg GAE g^{-1} DW
**Campagnano**	4.56^{ab}	108.31^{b}
**Castellammare**	3.30^{c}	74.30^{c}
**C3**	4.11^{bc}	106.66^{b}
**Grato 1**	3.32^{c}	70.46^{c}
**Orte1-GGR4 F17**	5.06^{a}	110.04^{b}
**Orte1-GGR5 F4**	4.57^{ab}	105.07^{b}
**Orte2-GGR11 F7**	5.11^{a}	126.63^{a}
**Orte2-GGR10 F8**	5.37^{a}	131.10^{a}
**CV**	17.90	20.92
p	***	***

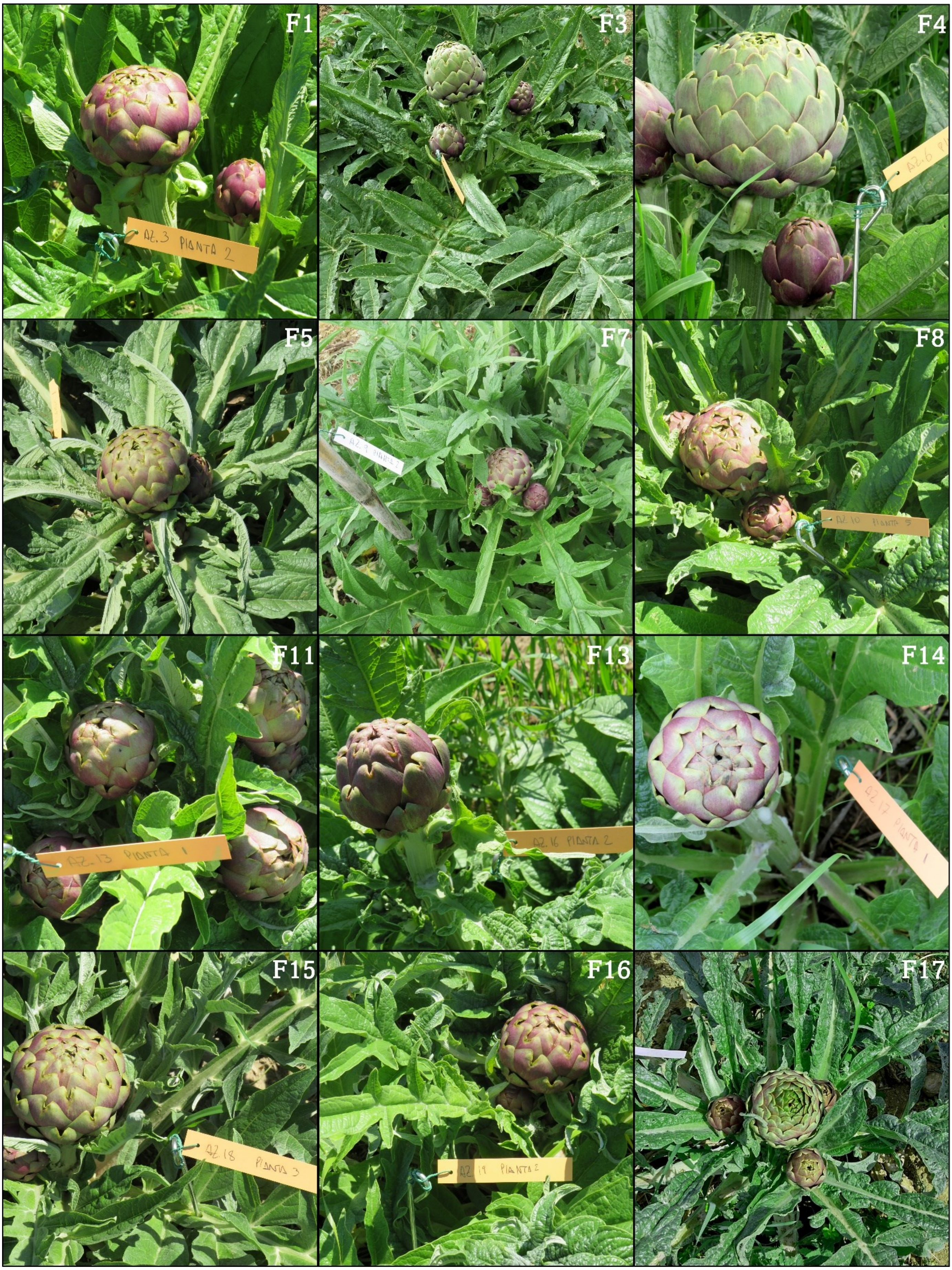

Figure S1. *Photos of some selected plants in twelve of the 20 smallholdings and family gardens in which the central and lateral flower heads are evident. The selected plants were marked with a stake bearing a tag in which was reported the plant number for each of the 20 smallholdings and family gardens.*

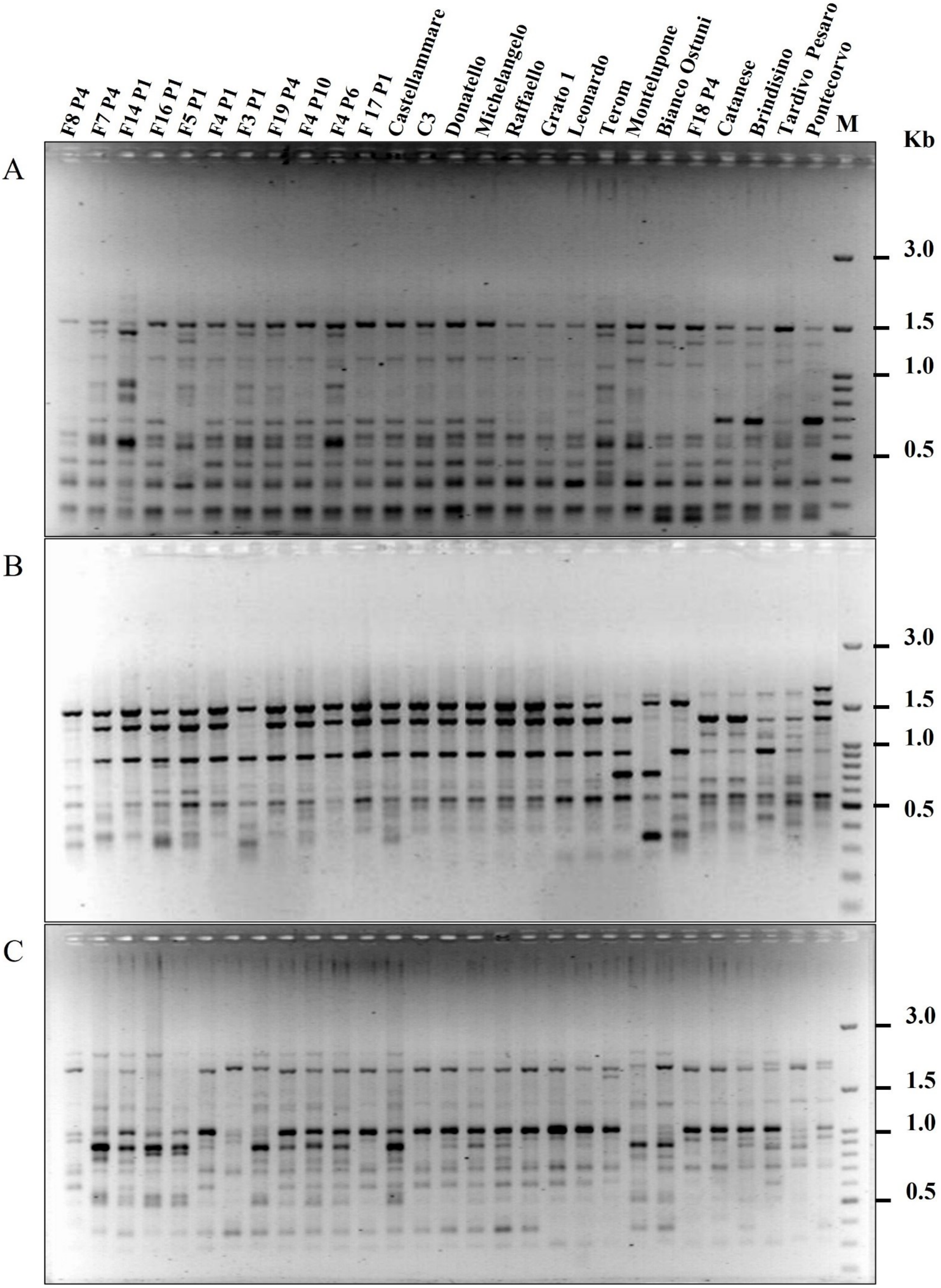

Figure S2. *Agarose gel electrophoresis of PCR products by three ISSR primers of 26 artichoke genotypes. A) ISSR 24, B) UBC 840, and C) UBC 855 primers.*

A

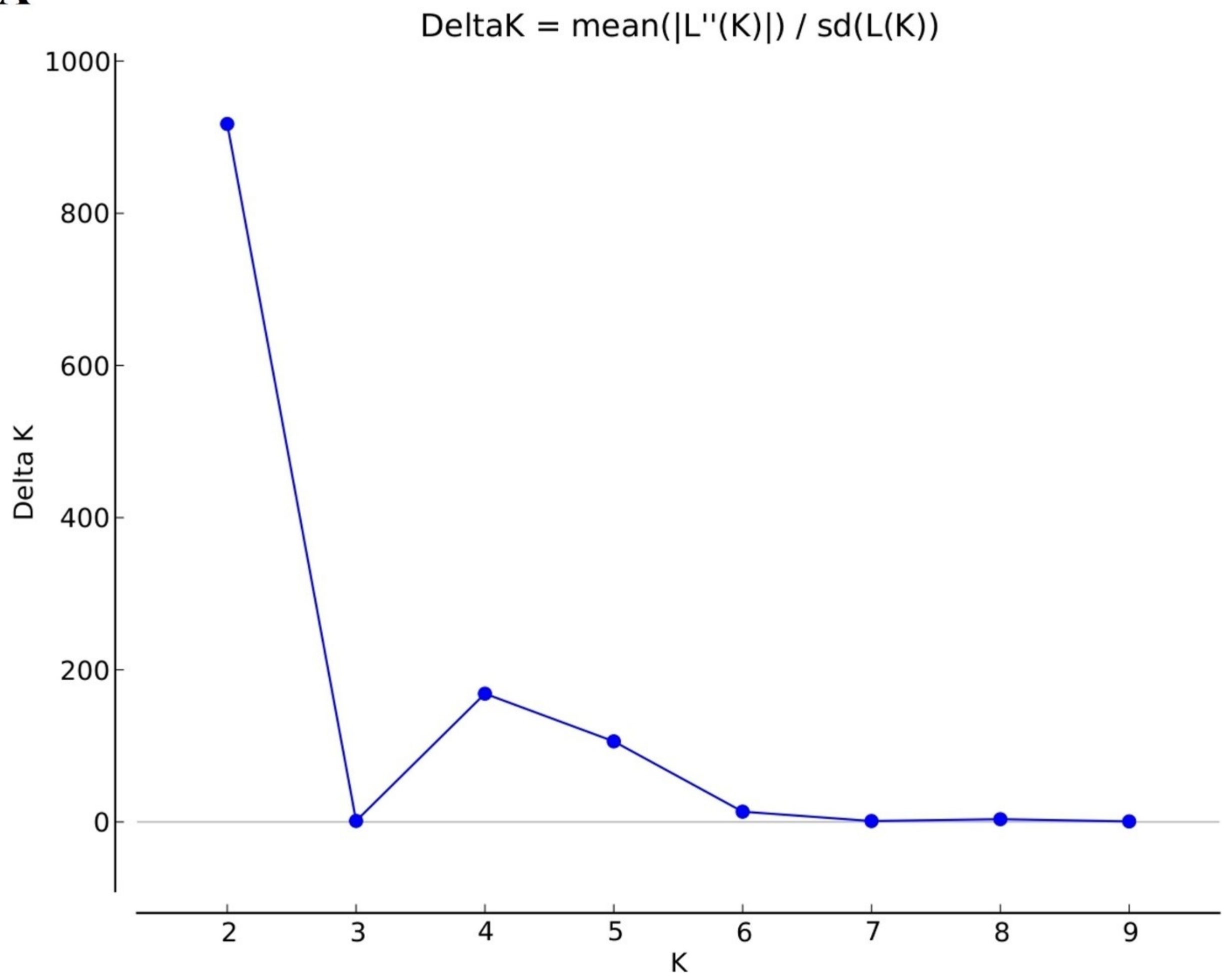

B

K	Reps	Mean LnP(K)	Stdev LnP(K)	Ln'(K)	|Ln''(K)|	Delta K
1	10	-2674.410000	0.276687	—	—	—
2	10	-1684.020000	0.737564	990.390000	676.670000	917.439581
3	10	-1370.300000	21.607818	313.720000	28.890000	1.337016
4	10	-1085.470000	0.707185	284.830000	119.260000	168.640373
5	10	-919.900000	0.862812	165.570000	91.400000	105.932702
6	10	-845.730000	13.868994	74.170000	187.640000	13.529460
7	10	-959.200000	186.312366	-113.470000	226.970000	1.218223
8	10	-845.700000	39.202466	113.500000	143.420000	3.658443
9	10	-875.620000	131.386570	-29.920000	96.840000	0.737062
10	10	-808.700000	61.727340	66.920000	—	—

Figure S3. *STRUCTURE analysis of 64 genotypes of the "Carciofo Ortano" landrace and eight landraces/clones of the "Romanesco" type. A) Estimation of the optimum number of clusters for the artichoke genotypes according to the Evanno's method. The graph displays the DeltaK [mean(|L''(K)|) / sd(L(K))] for each K value. B) Probabilities means of simulation models employing dissimilar K value*
